# The Generation of Involuntary Mental Imagery in an Ecologically-Valid Task

**DOI:** 10.3389/fpsyg.2021.759685

**Published:** 2021-10-21

**Authors:** Anthony G. Velasquez, Adam Gazzaley, Heishiro Toyoda, David A. Ziegler, Ezequiel Morsella

**Affiliations:** ^1^Department of Psychology, San Francisco State University, San Francisco, CA, United States; ^2^Neuroscape, Department of Neurology, University of California, San Francisco, San Francisco, CA, United States; ^3^Departments of Psychiatry and Physiology, University of California, San Francisco, San Francisco, CA, United States; ^4^Toyota Collaborative Safety Research Center, Ann Arbor, MI, United States

**Keywords:** involuntary imagery, unconscious processing, mental imagery, flanker task, semi-automated driving

## Abstract

Laboratory tasks (e.g., the flanker task) reveal that incidental stimuli (e.g., distractors) can reliably trigger involuntary conscious imagery. Can such involuntary effects, involving competing representations, arise during dual-task conditions? Another concern about these laboratory tasks is whether such effects arise in highly ecologically-valid conditions. For example, do these effects arise from tasks involving dynamic stimuli (e.g., simulations of semi-automated driving experiences)? The data from our experiment suggest that the answer to our two questions is yes. Subjects were presented with video footage of the kinds of events that one would observe if one were seated in the driver's seat of a semi-automated vehicle. Before being presented with this video footage, subjects had been trained to respond to street signs according to laboratory techniques that cause stimulus-elicited involuntary imagery. After training, in the Respond condition, subjects responded to the signs; in the Suppress condition, subjects were instructed to not respond to the signs in the video footage. Subjects in the Suppress condition reported involuntary imagery on a substantive proportion of the trials. Such involuntary effects arose even under dual-task conditions (while performing the *n*-back task or psychomotor vigilance task). The present laboratory task has implications for semi-automated driving, because the safe interaction between driver and vehicle requires that the communicative signals from vehicle to driver be effective at activating the appropriate cognitions and behavioral inclinations. In addition, our data from the dual-task conditions provide constraints for theoretical models of cognitive resources.

## Introduction

In *response interference* paradigms such as the Stroop task^1^ (Stroop, 1935) and the Eriksen flanker task (Eriksen and Eriksen, 1974), responses to a “target” stimulus are perturbed systematically by the incidental presence of “distractor” stimuli. In these tasks, interference, as indexed by error rates and response times (RTs), depends on the nature of the distractors. For example, in one variant of the flanker task (Eriksen and Schultz, 1979), subjects are first trained to press one button with one finger (e.g., the right index finger) when presented with the letter S or M, and to press another button with another finger (e.g., the right middle finger) when presented with the letter P. After training, the subjects are instructed to respond to targets that are “flanked” by distractors. They are instructed to respond to the stimulus presented in the center of an array (e.g., SSPSS, SSMSS, SSSSS, targets underscored) and to disregard the flanking stimuli, which are the distractors. In the original flanker task, subjects were instructed to “respond only to the letter in [a] location and to ignore any and all other letters” (Eriksen and Eriksen, 1974, p. 144). The shortest response times are found when the distractors are identical to the target (Eriksen and Schultz, 1979; van Veen et al., 2001). Slower RTs are found when the distractors are associated with a response that is different from that of the target (*response interference* [RI]) than when the distractors are different in appearance but associated with the same response (*stimulus interference* [SI]; Eriksen and Schultz, 1979; van Veen et al., 2001; Ridderinkhof et al., 2021)^1^.

Decades of research have focused on the behavioral effects of distractors in response interference paradigms. More recently, research has begun to focus on the subjective effects of these distractors. Regarding urges, for example, in “subjective” variants of the flanker task, “urges to err” are greater in the RI condition than in the SI condition or in a condition in which distractors are identical to the target (Morsella et al., 2009)^2^. Other effects in which distractors influence what enters consciousness involve, not urges, but the mental imagery (e.g., verbal imagery) that was associated with distractors during training (Bhangal et al., 2018; Bui et al., 2019; Cushing et al., 2019; Li et al., 2021). These effects reflect a kind of involuntary entry into consciousness (see also Scullin et al., 2021).

One concern regarding these subjective, distractor-elicited effects pertains to their ecological validity and robustness. Do these involuntary effects arise in real-world contexts in which the stimulus scene is complex and dynamic, for example, as occurs in driving? In real-world scenarios, critical objects are often not focal and not presented by themselves on a screen. Instead, these objects are nested within a complex field of view that is filled with other objects. Moreover, the field of view is usually, not static, but dynamic. A second question pertains to whether these involuntary effects are robust enough to arise under dual-task conditions, as when there is competition among mental representations. According to some theorists (Exner, 1879; Ach, 1905/1951; Woodworth, 1939; Neely, 1977; Gollwitzer, 1999; Hommel, 2000; Cohen-Kdoshay and Meiran, 2007, 2009), these involuntary effects should be detectable to some extent even during dual-task conditions. If this is the case, then theoretical accounts concerning cognitive resources must take such findings into account. We assume that the brain mechanisms that generate conscious mental imagery consume at least some cognitive/neural resources. These representations, as fleeting as they may be, are an achievement of sophisticated neural activity. It is interesting to consider that, when such imagery is the outcome of an automatic association, or of something akin to the “prepared reflex” (Ach, 1905/1951; discussed below), it might arise even under dual-task conditions. Theories concerning cognitive resources need to account for observations in which high-level cognitions, as in mental imagery, are unperturbed by, for example, dual-task conditions. More generally, theories concerning cognitive resources must account for the many findings concerning the prepared reflex, findings that suggest that the neural machinery is often unperturbed by secondary tasks.

Because of these concerns regarding subjective variants of response interference paradigms, we developed an experimental paradigm involving distractor stimuli that, though occurring in a complex, dynamic, and ecologically valid context, could still be (a) capable of yielding some involuntary imagery and (b) capable of being coupled with a secondary task (for dual-task conditions).

### Introduction of the Navigation-Monitoring Task

**Figure 1:**
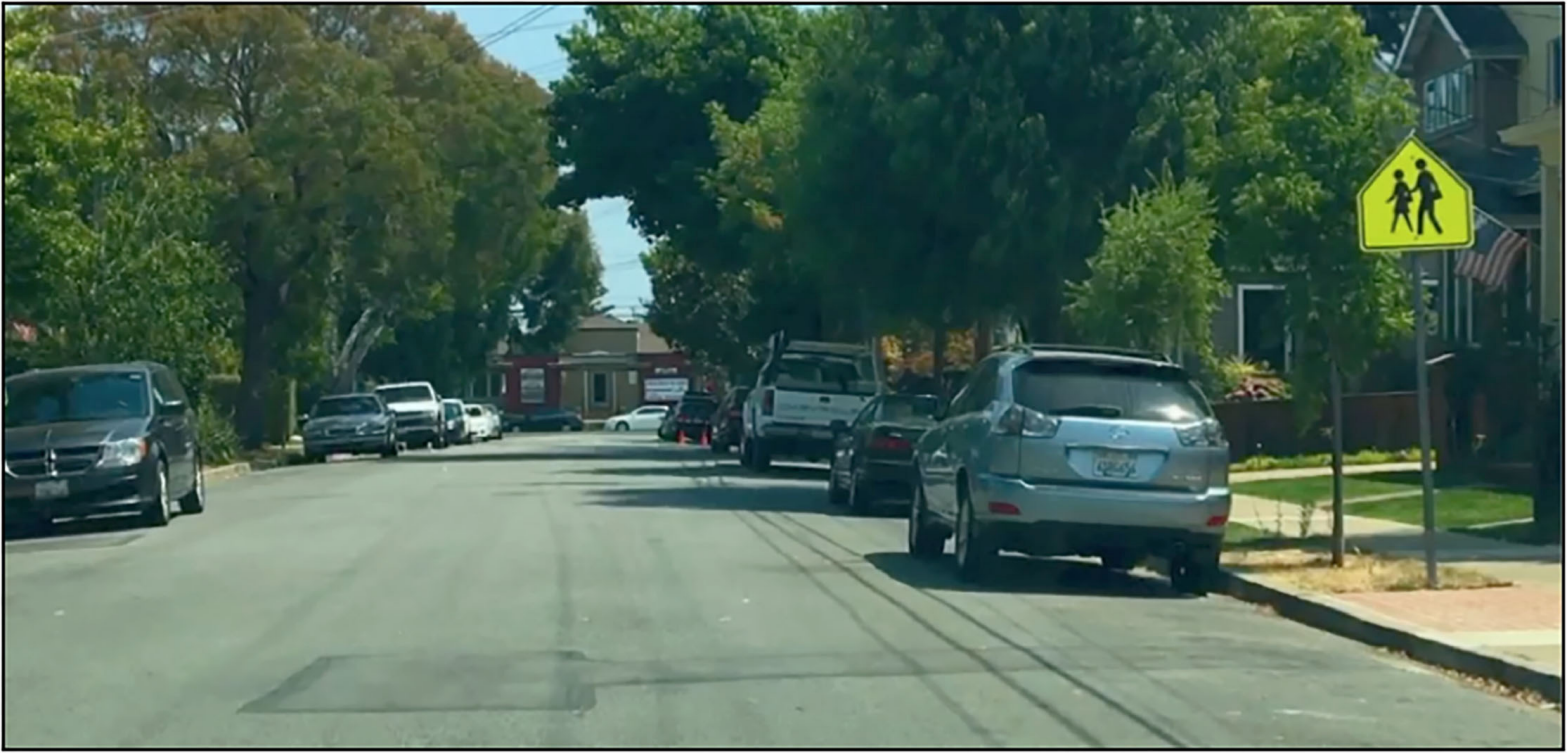
Sample image, taken from a 24-s film clip.

Through extensive piloting (*n* = 96), we developed a new kind of response interference paradigm, the *navigation-monitoring task*, which features a new kind of stimulus set. Distractor stimuli (real street signs) were embedded in actual video footage of the kinds of events that one would observe if one were seated in the driver's seat of a semi-automated vehicle (Figure 1). The video portrayed, from a first-person perspective, the vehicle approaching intersections, slowing down, speeding up, entering garages, etc. The stimulus videos were developed by us from over 36 h of actual driving footage. Basing our experimental paradigm on semi-automated driving renders our project ecologically valid and applicable to real-world challenges involving emerging transport technologies.

In the paradigm, subjects are first trained to detect and respond to certain street signs (e.g., a crosswalk sign) using a training procedure (*optimal response-signaling*, discussed below) that is based on laboratory techniques known to induce stimulus-elicited involuntary imagery (e.g., Allen et al., 2013; Gollwitzer, 2014). In the critical condition (Suppress), subjects are instructed to perform the navigation task, during which they monitor the navigation of the vehicle (e.g., press a button whenever the vehicle turns or merges). When performing this task, subjects are instructed to *not* respond to the critical signs in any way and to also *not* think of the response that, during training, was associated with each sign. As in the subjective variants of the flanker task (e.g., Morsella et al., 2009; Li et al., 2021), in this paradigm, experimenters can measure on a trial-by-trial basis the rates of involuntary imagery elicited by the distractors that are embedded in the video footage.

## Experiment

Our primary aim was to assess whether involuntary imagery could be elicited by the distractors in our newly developed, navigation-monitoring task, a task that is complex, dynamic, and ecologically valid. We sought to obtain substantive evidence that these involuntary effects can occur involuntarily at a reliable, substantive rate (no fewer than one trial out of five trials; based on Bui et al., 2019), even though the stimuli are complex, dynamic, and ecologically valid. We also assessed whether the subject could respond to the visual stimuli we developed with a good level of engagement, one that does not yield floor or ceiling effects. In addition, we assessed whether these effects are robust enough to be detectable under dual-task conditions. To this end, we coupled our paradigm with two well-established tasks: the *n*-back task (Kirchner, 1958) and the *psychomotor vigilance task* (PVT; Dinges and Powell, 1985; Jung et al., 2011). One hypothesis is that the dual-task condition would interfere with the mechanisms that generate involuntary imagery (Cho et al., 2014), thereby decreasing substantively the proportion of trials in which such imagery occurs. Given the automatic nature of involuntary imagery, and based on the findings of a previous study (Cho et al., 2014), in which involuntary, stimulus-elicited imagery arose even while subjects performed a secondary task (humming in one's head but not aloud), we hypothesized that the secondary task would not decrease the rates of involuntary imagery substantively. We also took the opportunity to assess another manipulation: whether involuntary imagery still arises when the number of signals that are learned are, not three, but double that number, that is, six signals.

We employed a design that was 2 (Respond [to road signs] or Suppress [responses to road signs]) × 2 (three signals or six signals) × 3 (No Multi-Tasking, *n*-back, or PVT). (The third factor, Multi-Tasking, was the only factor that was held within-subjects; the other factors were between-subjects factors).

### Prediction

We predicted that, despite the complex, ecologically-valid, and dynamic nature of the stimulus scene, and despite the dual-task conditions, these involuntary, stimulus-elicited effects on consciousness will be detectable (with the rate of detection being significantly above zero) and will occur at a reliable, substantive rate (no fewer than one trial out of five trials; based on Bui et al., 2019).

## Method

### Subjects

Eighty-four (21 per cell of the 2 × 2 × 3 design) San Francisco State University students (*M*_age_ = 20.24, *SD*_age_ = 4.54, females = 57) participated in a 120-minute session for $20. The Institutional Review Board at San Francisco State University approved the involvement of human subjects in our project. Prior to participation in the study, all subjects provided written and verbal consent. The sample size (*n* > 10) was based on the effect size (Cohen's *d* [on raw proportions] = 1.72; Cohen's *h* [on raw proportions] = 1.44; Cohen's *d* [on arcsine transformations of the proportion data] = 1.38), *SD* (0.25), and other aspects of a previous project (Cho et al., 2016) that, similar to the present project, was designed to illuminate the boundary conditions of involuntary entry into consciousness. To determine the sample size, we used the program G^*^Power 3 (Faul et al., 2007). The input parameters were: Cohen's *d* = 1.72, one sample *t*-test, tails = one, power = 0.95, and α = 0.05. The output parameters were: non-centrality parameter = 4.21, critical *t* = 2.02, and actual power = 0.97.

### Stimuli and Apparatus

The video stimuli were presented on a black background of a 50.8 cm Apple iMac computer monitor (Apple iMac 7, 1 A1224) with a viewing distance of ~48 cm. Stimulus presentation and data collection were controlled by PsyScope software (Cohen et al., 1993). Subjects inputted their responses to questions and instructions by computer keyboard. All questions and instructions were presented in white or otherwise colored 44-point Arial font; all fonts were displayed on a black background. (The “Ready?” prompt was 40-point Arial font; the “rest time” prompt between the critical trials was 24-Arial font.)

We used an additional iMac keyboard as the input device for the button presses. Hence, the Apple iMac computer was connected to two keyboards. This secondary keyboard was not used by the subject for typing responses but only for responding to the signs. We covered the eight input keys with the overlays having the appropriate colors. Specifically, the tab key on the left of the standard keyboard served as the “Black key,” and the / key served as the “White key,” which was used during the multi-tasking conditions (explained below). We strove to preserve the spatial layout of a button box (Response Box; ioLab Systems; UK) that was used only during piloting. Thus, the remaining designated keys were as follows. Red = Z key; Orange = D key; Yellow = T; Green = U; Blue = K, Purple = period.

The video presented to subjects was actual footage of the kinds of events that one would observe if one were seated in the driver's seat of a semi-automated vehicle. The video portrayed, from a first-person perspective, the vehicle approaching intersections, slowing down, speeding up, etc. To have ecological validity, the footage retained the unexpected motions of the vehicle (e.g., when driving over a pothole) and unexpected visual phenomena (e.g., transient sun glare). Each video was composed of a series of short clips, some of which presented the critical, trained stimuli. The subject experienced the clips as a quasi-continuous movie of a vehicle navigating on roads and city streets. Most of the footage was of city streets, suburban neighborhoods, and highways in the cities of Burlingame, Oyster Point, South San Francisco, and San Francisco.

Each experimental session included the presentation of 60 critical clips, which included signs from the training session. The ~120 min of final, color video footage that was presented to subjects was developed from over 36 h of raw footage obtained with an iPhone 6S camera mounted on the dashboard of a Lexus RX 350 (2014). The frame of the video clip was adjusted so that the hood of the vehicle would not be visible (Figure 1). The final footage that was selected had to satisfy many criteria, including that the clip presenting a sign did not present any of the other critical signs, that the weather conditions rendered the stimuli perceptible, and that the clip did not present any stimulus that would interfere with perceiving the critical sign or with responding to the critical sign.

Presented on a black background, each of the selected, final clips (33 cm wide × 18.5 cm high, with a viewing angle of 37.94° × 21.81°) was extensively video-edited, by hand tuning, through the software iMovie to increase the speed of the clip, increase contrast, decrease exposure, reduce brightness, increase the color saturation of the signs, and adjust other properties, so that all of the clips, though naturalistic and ecologically valid, were as uniform as possible.

The critical clips, which included the presentation of the trained signs, were composed of three parts. First, there was 20 s of footage in which none of the critical signs or control (untrained) signs were presented. After these 20 s, there was a one-second clip that presented footage of the vehicle driving toward a critical, trained sign or a control stimulus, with the signs appearing in their natural context (Figure 1). In these one-second segments, the sign stimuli were not ever occluded and were clearly perceptible. After the presentation of the stimulus, there were 3 s of extra footage. Because stimulus-elicited imagery could arise during this time, we avoided having novel, “attention-grabbing” stimuli presented during this span. Instead, we presented some of the uneventful footage taken from the 20 s of footage preceding the presentation of the sign. Subjects never saw the same entire critical clip twice, but they did sometimes see, more than once, footage taken from the same geographical region or, in very few cases, see, parts of a video sequence repeated in another critical clip. When developing the complex stimulus (the video footage), we strove for it to be ecologically valid and challenging, so that the detection of signs embedded in the footage would not be too easy or too difficult.

In each session, in addition to the 60 critical clips, subjects were presented with 60 “filler” clips, which, in terms of their content, resembled the critical clips in all respects but did not present any of the critical signs (i.e., the signs associated with training). These filler clips varied in duration, with most being 10, 16, or 20 s. This variability in duration was intended to diminish the predictability of the timing of the events composing the session.

In the training session (see details below), we presented only a single static frame of a real sign in its actual setting (Figure 1). This static image (a photograph) was culled from the raw footage. For the training session, there were 10 unique photographs per sign, and subjects never saw these stimuli again during the critical trials.

### Procedures

#### The Navigation-Monitoring Task

Instructions were presented *via* computer screen. The first instruction to subjects was the following. “*You will see video taken from a vehicle that is driving. Press the black button when you see the vehicle turning left or right or merging left or right. That is, press the black button whenever the vehicle turns or whenever it merges to another lane. You will be doing this throughout the whole session. Press RETURN to continue*.”

Afterwards, subjects were instructed, “*At the beginning of each trial of the driving task, your hands must be in ‘rest position,’ which is having the palm of your right hand resting and having your left hand ready to press the black button. The experimenter can show you how to put your hands in this rest position. Press RETURN to practice this task*.” Subjects then viewed a 10-second clip in which the vehicle made a right turn. Subjects were instructed to press the black button as soon as they noticed the car turning or merging into another lane. This video footage was never presented again during the experimental session. The black button was actually a dummy button. During the experimental session, the experimenter made sure that subjects were pressing the dummy button during the task. We were not able determine the accuracy of each of these responses. The depressing of the black button did not cause any modification in the computer program or data output.

**Figure 2:**
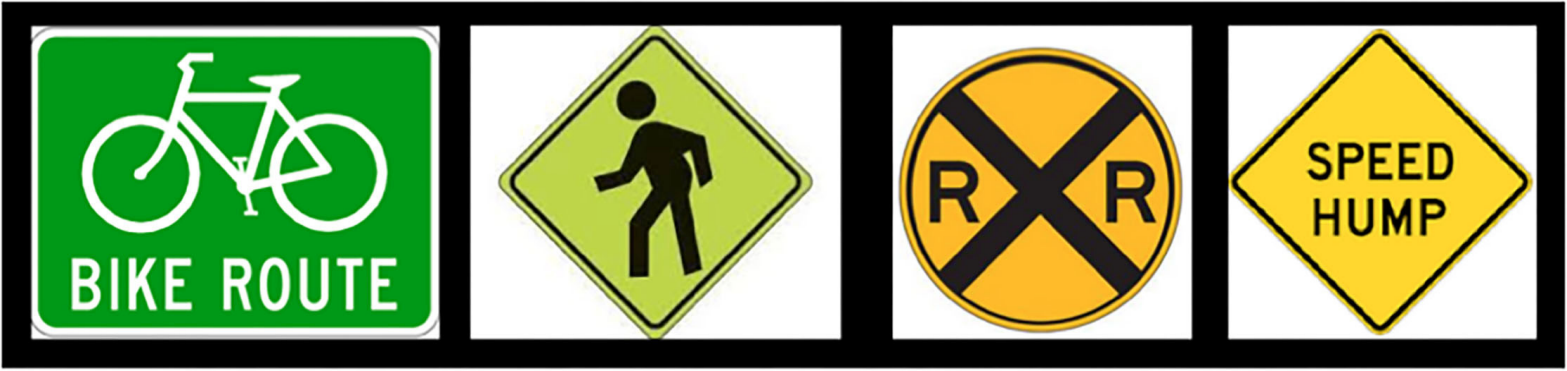
Sample stimulus signs.

**Figure 3:**
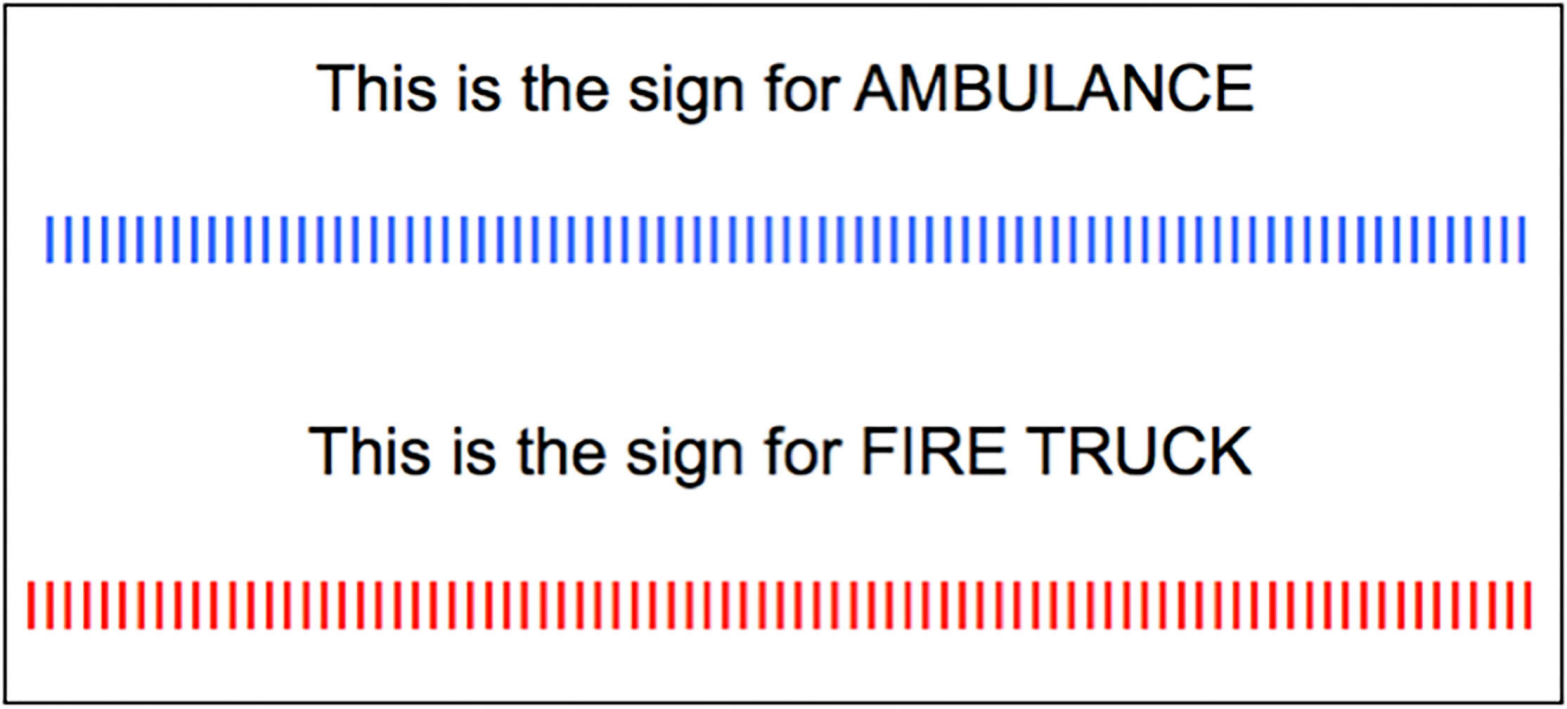
The manner in which subjects were presented with the Ambulance and Fire Truck signs. The Ambulance sign (presented in blue [top]) and the Fire Truck sign (presented in red [bottom]).

After this training event, subjects were told, “*For the upcoming task, you need to be familiar with the following street signs*.” Except for the Ambulance and Fire Truck signs, for each kind of sign, subjects were presented with an array of four versions of it (e.g., a Crosswalk sign depicting one person walking and a Crosswalk sign depicting two people walking). The signs presented in the arrays were not from photographs but were stylized, color diagrams, the kind of depiction that one would see in a driver's manual (Figure 2). Each array was presented for 5 s. The Ambulance signs and Fire Truck signs were presented by themselves (Figure 3).

#### Training Session

During training, subjects learned certain responses to the stimuli that would later be presented as distractor stimuli in the navigation-monitoring task. This was based on the procedures of subjective variants of the flanker task (e.g., Morsella et al., 2009; Li et al., 2021). As in the training for the flanker task, during the acquisition of these response codes, it is beneficial for the subject to experience the actual consequences of the self-generated action (Guthrie, 1935; Hommel, 2000, 2009; Hommel et al., 2001, 2016; Olsson and Phelps, 2004; Samaha et al., 2013; Chen et al., 2014). To maximize the effects of our training session, the instructions for performing the stimulus-elicited action were of the form, “If I encounter the situation *X*, then I will perform the response *Y*” (Gollwitzer, 2014). As Gollwitzer (2014) notes, “Whereas goal intentions merely specify a desired future behavior or outcome, the if-component of an implementation intention [prepared reflex] specifies when and where one wants to act on this goal (i.e., a certain situational cue), and the then-component of the implementation intention specifies the response that is to be initiated” (p. 306). Prior research (e.g., Gollwitzer, 2014), and our piloting, suggests that this is an effective way to induce involuntary, stimulus-elicited effects. The acquisition of the stimulus-response contingency through such verbal instructions, without extensive training, has been characterized as something akin to the acquisition of a “prepared reflex” (Exner, 1879; Ach, 1905/1951; Woodworth, 1939; Gollwitzer, 1999; Hommel, 2000; Cohen-Kdoshay and Meiran, 2009; Cole et al., 2013; Pereg and Meiran, 2019). The term reflects that the effects of this form of knowledge acquisition resemble, remarkably, those of involuntary stimulus-response links. The acquisition of these stimulus-response contingencies require very few trials (e.g., less than 10 trials). See recent review of the neural correlates of such a rapid form of learning (also called *Rapid Instructed Task Learning* [RITL] in Cole et al., 2013; Pereg and Meiran, 2019). Again, the involuntary effects resulting from this kind of learning are proposed to be robust enough to be detectable under dual-task conditions (Exner, 1879; Ach, 1905/1951; Woodworth, 1939; Gollwitzer, 1999; Hommel, 2000; Cohen-Kdoshay and Meiran, 2009; Cole et al., 2013; Pereg and Meiran, 2019).

During stimulus-response acquisition (10 trials for each signal) for the experimental condition, subjects learned to associate certain stimuli/signals with certain specific responses. Training included (a) the actual experience of the action-effect following one's self-generated action, and (b) instructions in the form of “If I encounter the situation *X*, then I will perform the response *Y*,” to induce a “prepared reflex” or “implementation intention” (Gollwitzer, 1999, 2014). When combined in one training session, these components could be construed as yielding an optimal form of stimulus-elicited, response-signaling (optimal response-signaling, for short).

For training, subjects learned to respond to the signs by virtue of the instructions presented below. Subjects were instructed to read these instructions aloud. After reading each set of instructions, subjects responded as instructed to a photograph that presented the sign for 3.5 s. They repeated this sequence of reading the instruction and responding to a stimulus ten times. The stimulus consisted of a single static frame of a real sign in its actual context. This static image (a photograph; Figure 1) was taken from the raw footage. There were 10 unique photographs per sign and subjects never saw these stimuli again during the critical trials. Using different stimuli on each of the 10 training trials diminishes the effects of stimulus-specific habituation (Bhangal et al., 2016) and also increases the likelihood of “stimulus generalization” across the entire stimulus class, so that training-based effects will arise for all school zones, for example.

For the crosswalk sign, the instructions were “*When you see a CROSSWALK sign, say ‘Yellow Yield’ aloud and press the YELLOW button. It is important that you respond as fast and as accurately as possible. Your hands must be in ‘rest position.’ Press G when you are ready to see the sign and respond to it*.” (See instructions for all signs in the Appendix).

In one condition, subjects were trained on only three signs of the six possible signs. The order of presentation of each of the three signs was random. Half of the subjects were trained in this way for Bike Lane, Speed Bump, and Fire Truck (Regimen 1). The other half of the subjects were trained in this way to respond to Crosswalk, Railroad Crossing, and Ambulance (Regimen 2). In each group of signs, there was one sign pertaining to an event that required for attention to be deployed to an upcoming event that would be occurring (usually) straight ahead, on the center of the road (Speed Bump and Railroad Crossing) and perpendicular to the direction of the vehicle; one sign that pertained to an event requiring also attention to be deployed to the right side of the road (Bike Lane and Crosswalk); and one event that pertained to sirens and emergency vehicles (Ambulance and Fire Truck; Figure 3). In short, each regimen contained one of each kind of sign.

Before the commencement of the critical trials, subjects read, “*You will now perform the driving task. Again, you will see video taken from a vehicle that is driving. Press the black button when you see the vehicle turning left or right, or merging left or right. That is, press the black button whenever the vehicle turns or merges into another lane. Respond as quickly and as accurately as you can*.” This was followed by a screen that presented, “Ready? Please be in ‘rest position’ and press the black button when ready?”

The 60 critical trials per session were presented as three blocks, with each block having 20 trials of randomly selected critical video clips. Each block was randomly coupled with one of the three Multi-Tasking conditions (each described below). Across the three blocks, each kind of sign (Bike Lane, Speed Bump, Crosswalk, Railroad Crossing, Ambulance, and Fire Truck) appeared 10 times. Within each block, the “embedded” signs (Bike Lane, Speed Bump, Crosswalk, and Railroad Crossing) appeared on 12 randomly selected trials of the 20 trials, and the unembedded signs (Ambulance and Fire Truck) appeared on eight randomly selected trials of the 20 trials. The subject never saw a given critical stimulus more than once.

#### The Suppress Condition

Before the critical trials of the Suppress condition, subjects (*n* = 42) were presented with the following. “*IMPORTANT: During the task, please DO NOT respond to any of the signs. Although you were presented with information about how to respond to the signs, you must NOT respond to any of the signs. Also, try to NOT think of the response you learned to any of the signs. However, if you do happen to think of the response to any of the signs in the video, then please report such thoughts when you are asked about them at the end of the given clip*.” After each trial of the Suppress condition, subjects reported about involuntary verbal imagery, based on instructions given before the beginning of the critical trials.^3^

Before the critical trials, subjects read, “*You are now ready to perform the task. There will be three blocks of trials, with each block taking around 15 min. IMPORTANT: If you have any questions about the task or about the nature of verbal imagery, please ask the experimenter. Press RETURN when ready to begin the task*.”

After each critical trial, subjects answered the following questions. ***1***. “*Did you just experience any verbal imagery? That is, did you experience any verbal imagery during the last moments (that is, the last 5 sec) of the video? (Yes or No?),”* ***2***. “*If you did experience any verbal imagery, please type the words you experienced,”* ***3***. “*If you did experience verbal imagery, did the words come to mind immediately? (Yes or No?),”* ***4***. “*During these last moments (5s) of the video clip, how strong was the urge to press a button?”* Subjects responded to the last question using an 8-point scale, in which 1 signified “almost no urge” and 8 signified “extremely strong urge” (based on Morsella et al., 2009).

#### The Respond Condition

Before the critical trials of the Respond condition, subjects (*n* = 41) received the following instructions. “*You will now perform the driving task. Again, you will see video taken from a vehicle that is driving. Press the black button when you see the vehicle turning left or right, or merging left or right. That is, press the black button whenever the vehicle turns or merges into another lane. Respond as quickly and as accurately as you can. IMPORTANT: During the task, please respond to the signs as instructed*.”

The experimenter clarified responses should consist of just the button presses and that the vocal responses were no longer necessary. The continued execution of the vocal responses would have introduced several practical and experimental-design problems, including that, over the course of the trials, the vocalizations would induce a form of training for the control signs.

#### Manipulations of Memoranda and of Multi-Tasking

For the between-subjects factor Memoranda, we manipulated the number of trained signals (3 or 6 signals). When the level of this factor was 3, we took the opportunity to compare the effects of trained signs (three in number) vs. untrained signs (three in number), which were not associated with any form of training.

For the within-subjects factor Multi-Tasking, in one of the experimental conditions, subjects performed, while carrying out the navigation-monitoring task, a second task that taxed cognitive control and working memory: An auditory version of the *n*-back (*2*-back) task (Kirchner, 1958) that presents sequences of numbers auditorily (Perlstein et al., 2003; based on Goncalves and Mansur, 2009). (All secondary tasks were auditory in nature, so that the stimuli would not occupy the visual buffer that was employed for the navigation-monitoring task; Baddeley, 2007). A *2*-back condition of the *n*-back task occurred on a block of 20 consecutive trials, with the total number of critical trials in the session being 60 trials. Each sequence occupied the span of the 24 s of the critical trials, with 3 s of silence between each of the six auditory stimuli (each auditory stimulus occupied 1 second of the 24 s span). Eight of the 20 trials presented “hit” trials, in which a heard number was heard before (the number heard before the previous number: e.g., 5 4 2 1 7 1, with 1 being the target). Targets were presented in the third, fourth, fifth, and sixth positions of the sequence. To report a hit, subjects used the “white key” (which was the / key on the keyboard). In the control trials (*n* = 12), there were no numbers repeated in the sequence in this way (e.g., 5 4 2 3 7 1). The 20 trials of the *n-*back were presented in random order. For various practical reasons (e.g., variable trial length), no *n*-back stimuli were presented during the filler trials. Before the block of trials, the subjects were presented with instructions for performing the auditory *2*-back (based on Perlstein et al., 2003; Goncalves and Mansur, 2009).

In another condition, which, too, consisted of a block of 20 consecutive trials, subjects performed a task that taxes sustained attention (a task based on the auditory version of the *psychomotor vigilance task* [PVT]; Dinges and Powell, 1985; Jung et al., 2011). For this task, subjects were instructed to, in addition to carrying out the many responses for the navigation-monitoring task, press a button as soon as they heard an auditory signal (a beep) during the 24 s of the critical trials. For various practical reasons (e.g., the variable lengths of the filler trials), no signals were presented during the filler trials. This auditory stimulus was presented only once per trial. The beep sounded at 5, 6, 6.5, 7, 8, 9, 10, 11, 12, 13, 14, 15, 15.5, 16, 17, 17.5, 18, 19, and 20 s.

**Figure 4:**
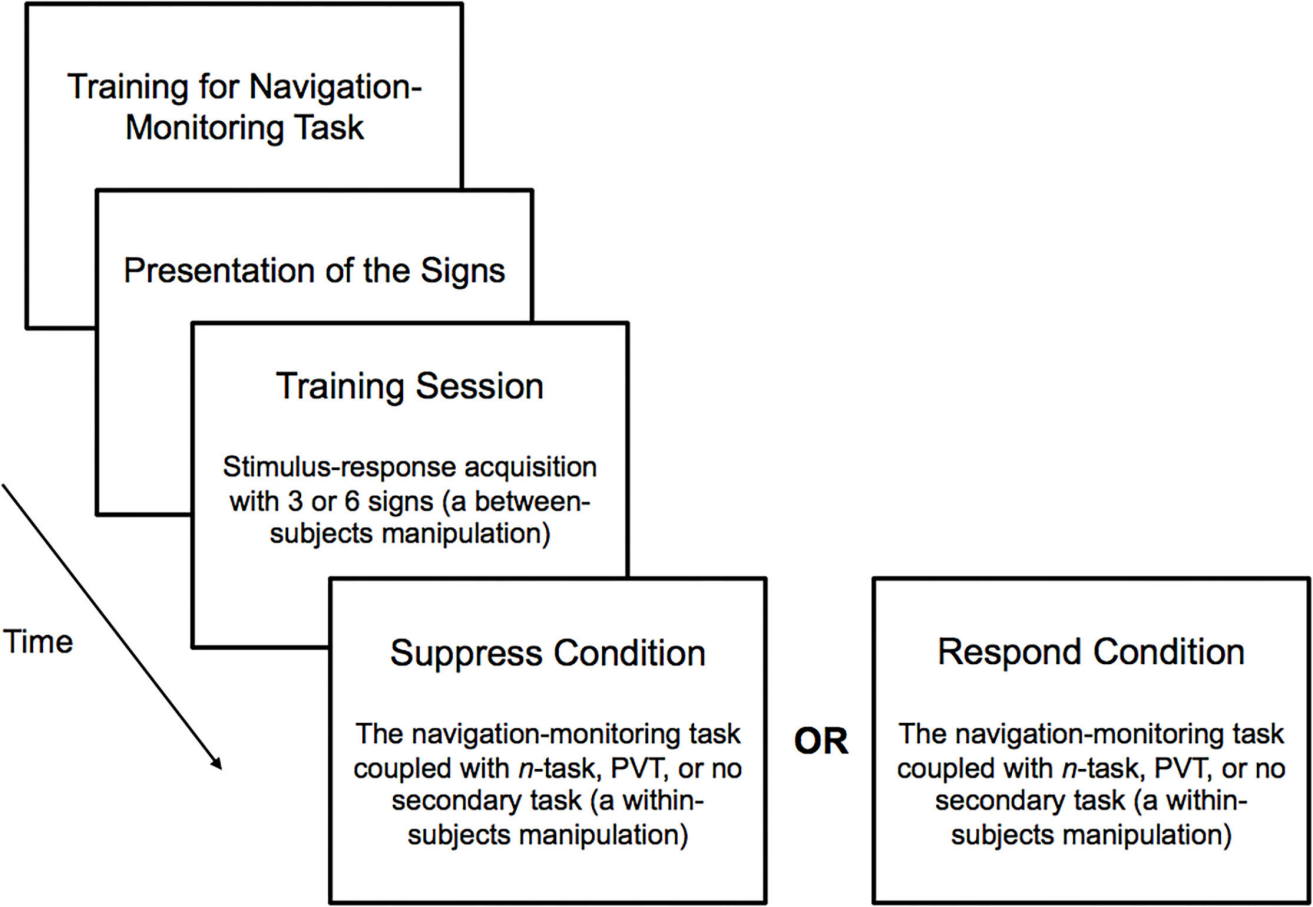
Schematic depiction of sequence of events composing the experimental session.

For the sake of comparison, there was a block of 20 consecutive trials in which subjects performed the navigation-monitoring task without any secondary task (i.e., a “No Multi-Tasking” condition). The order of presentation of the three conditions composing the factor Multi-Tasking was fully counterbalanced across subjects. See Figure 4 for a depiction of the sequence of events composing the experimental session. The data from one subject from the Respond condition were excluded from analysis because this subject performed only 20 trials of the 60 trials and was not following instructions.

#### Dependent Measures and Data Analysis Plan

The dependent measures of interest involved subjects' experience of involuntary imagery in the Suppress condition. The primary dependent measure was the mean proportion of trials in which subjects reported that they experienced involuntary imagery. We simply divided the number of trials in which there was imagery by the number of trials in that respective block of trials. For example, if *Subject 5* had imagery on 10 trials out of the 20 trials composing the PVT block, then the dependent measure for that condition, for that subject, would be 0.50. These proportions were treated as a continuous variable in the same manner that mean accuracy rates or mean error rates are treated as continuous variables. We conducted one-sample *t*-tests to assess whether these mean proportions were significantly different from zero. We also conducted an ANOVA to assess whether the memoranda manipulation (i.e., for three signs or six signs) and the three multi-tasking conditions had differential effects on the proportions of involuntary imagery. Because proportions are sometimes not normally distributed, we also performed these inferential statistics on the arcsine transformations of the proportion data. Arcsine transformations are often used to statistically normalize data that are in the form of proportions. All the significant effects presented below, concerning rates of involuntary imagery, were also found with arcsine transformations of the proportion data.

Another dependent measure was the mean proportion of trials in which subjects reported that the imagery was immediate. For this measure, we conducted an ANOVA to assess whether the two memoranda conditions and the three multi-tasking conditions had differential effects on the immediacy of the involuntary imagery.

Another dependent measure was subjects' trial-by-trial urges to err, which was based on an 8-point scale, in which 1 signified “almost no urge” and 8 signified “extremely strong urge.” For this measure, we conducted an ANOVA to assess whether the two memoranda conditions and the three multi-tasking conditions had differential effects on the mean urges.

## Results

### Involuntary Imagery

Computer malfunctions led to the loss of 37 (0.01%) of 2,520 trials in the Suppress condition. The mean removal of trials per subject was <1 trial (*M* = 0.88). These malfunctions caused some critical trials to appear more than once per session. Data from these repeated trials were removed from all analyses.

**Figure 5:**
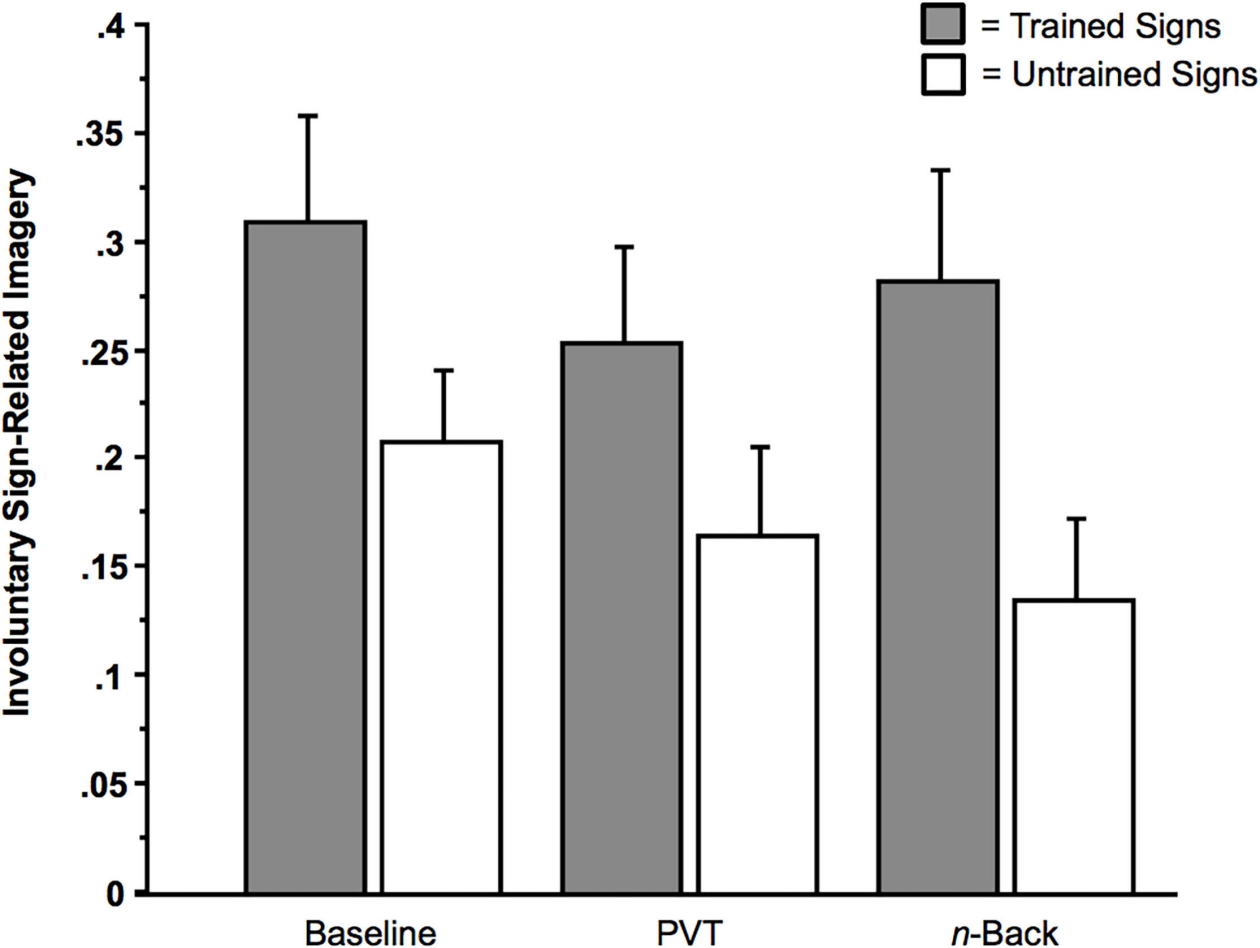
Sign-related involuntary imagery as a function of Sign Type (Trained vs. Untrained [Control]) and Multi-Tasking (None [Baseline], Psychomotor Vigilance Task [PVT], or *n*-back). Error bars indicate *SE*s.

One aim of our analysis was to ascertain whether involuntary imagery arose in a substantive manner by the signs that were embedded in the video footage (e.g., the Speed Bump, Railroad Crossing, Crosswalk, and Bike Lane). Any effects in the Suppress condition are noteworthy because subjects were instructed to not respond to any of the signs. Despite the intentions of the subjects, involuntary imagery arose in response to the presentation of the street signs. This *involuntary sign-related imagery* was defined as the involuntary imagery of the color, name, or verbal associate of the sign (e.g., the “move” in “red move”). In response to the second question, there was involuntary imagery reported on a substantive proportion of the critical trials, as illustrated in the baseline, No Multi-Tasking condition (*M*_Proportion of Trials_ = 0.31 of 20 trials, *SD* = 0.26, *SE* = 0.05, Range = 0 to 0.90). This mean proportion was significantly different from zero, *t* (27) = 6.31, *p* < 0.0001 (Cohen's *h* = 1.18), as were the other two mean proportions from the trained-sign conditions, that is, the *n-*back condition (*M*_Proportion of Trials_ = 0.28 of 20 trials, *SD* = 0.28, *SE* = 0.05, Range = 0 to 0.83) and the PVT condition (*M*_Proportion of Trials_ = 0.25 of 20 trials, *SD* = 0.23, *SE* = 0.04, Range = 0 to 0.78), *t*s (27) > 5.37, *p*s < 0.001 (Cohen's *h* = 1.12 [*n*-back], Cohen's *h* = 1.05 [PVT]). The mean proportions presented in Figure 5 that stemmed from the Untrained conditions, too, were significantly different from zero, *t*s (27) > 4.07, *p*s < 0.001.

Consistent with the hypothesis that these involuntary effects are robust enough to be detectable under dual-task conditions, the factor of Multi-Tasking (PVT, *n*-back, or None [baseline]) had no effect on the rate of occurrence of involuntary imagery. For example, as illustrated in Figure 5, in a 2 × 3 ANOVA with the within-subjects factor Sign Type (Trained or Untrained), and the within-subjects factor Multi-Tasking (None [Baseline], PVT, or *n*-back), there was only one main effect: a main effect of Sign Type on *involuntary sign-related imagery* (the color, name, or verbal associate of the sign), in which trained signs yielded more involuntary imagery than did untrained signs, *F*(1, 27) = 12.64, *p* = 0.0014 (η_*p*_^2^ = 0.32). There were no other significant main effects or interaction effects between the factors, *F*s < 2.75, *p*s > 0.05.

The same results are found with the following analysis, in which, for the sake of thoroughness, we included the contrast between the two training regimens for the condition in which subjects were trained on only three signs. (Some subjects received training for the three signs of Bike lane, Speed Bump, and Fire Truck [Regimen 1], while other subjects received training for the signs of Crosswalk, Railroad Crossing, and Ambulance [Regimen 2]). In this 2 × 2 × 3 ANOVA, with the within-subjects factor Sign Type (Trained or Untrained), the between-subjects factor Regimen (1 or 2), and the within-subjects factor Multi-Tasking (None [Baseline], PVT, or *n*-back), there was only one main effect: a main effect of Sign Type on *involuntary sign-related* imagery, in which trained signs yielded more involuntary imagery than did untrained signs, *F*(1, 26) = 12.28, *p* = 0.002 (η_*p*_^2^ = 0.32). There were no other significant main effects or interaction effects between the factors, *F*s < 2.76, *p*s > 0.05.^4^

Even when subjects were trained on six signs, there was involuntary sign-related imagery on a substantive proportion of the trials. This was the case for the baseline condition (*M*_Proportion of Trials_ = 0.36 of 20 trials, *SD* = 0.24, *SE* = 0.06, Range = 0 to 0.75), the PVT condition (*M*_Proportion of Trials_ = 0.31 of 20 trials, *SD* = 0.27, *SE* = 0.07, Range = 0.05 to 0.82), and the *n*-back condition (*M*_Proportion of Trials_ = 0.29 of 20 trials, *SD* = 0.19, *SE* = 0.05, Range = 0 to 0.70). Each of these three mean proportions was significantly different from zero, *t*s (13) > 4.33, *p*s < 0.001 (Cohen's *h*_Baseline_ = 1.29; *h*_PVT_ = 1.18; *h*_*n*__-back_ = 1.14).

**Figure 6:**
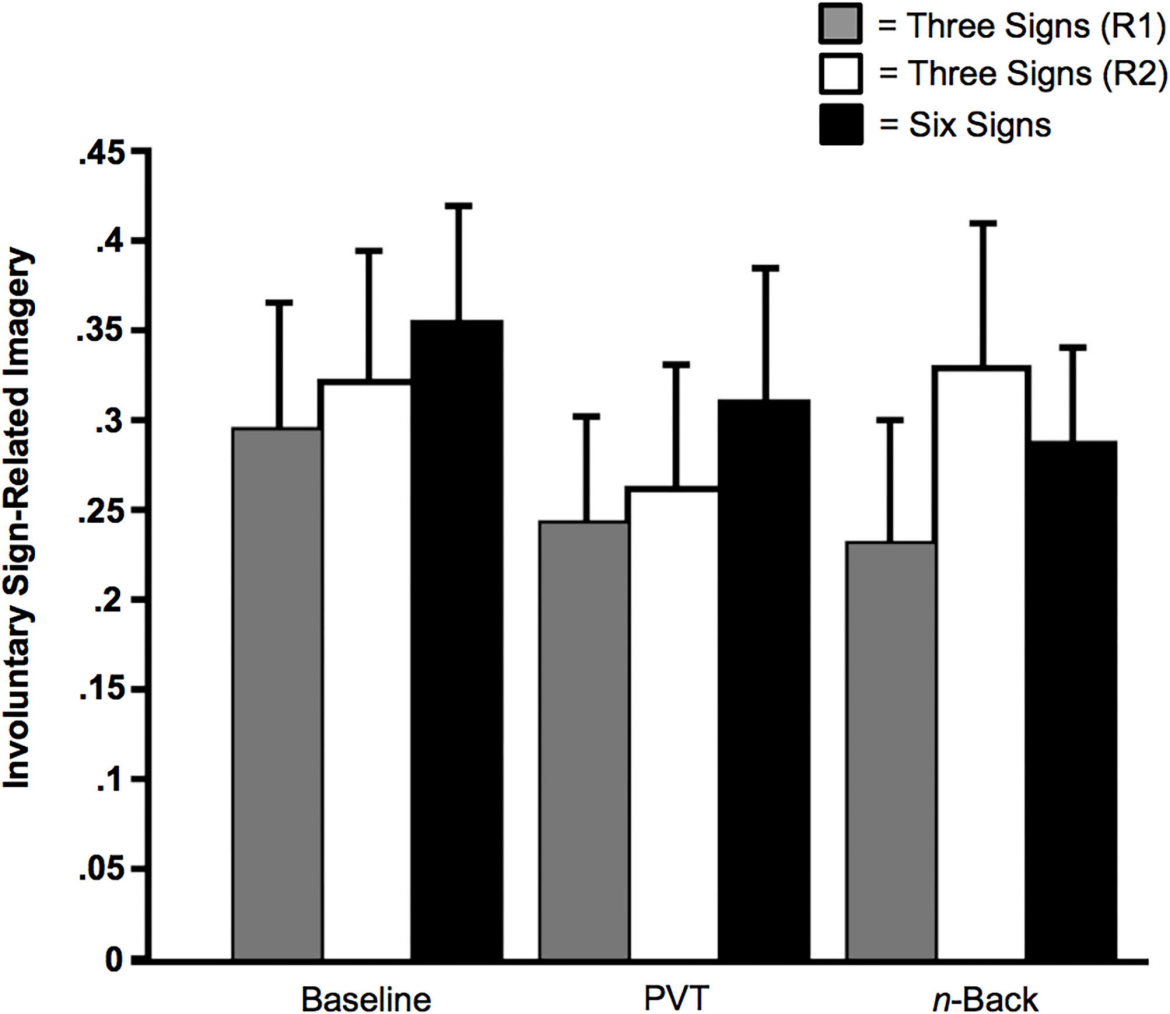
Sign-related involuntary imagery as a function of Memoranda (three signals or six signals) and Multi-Tasking (None [Baseline], Psychomotor Vigilance Task [PVT], or *n*-back). The solid black bars indicate subjects who received training for all six signs. The gray bars indicate subjects who received training for the three signs of Bike Lane, Speed Bump, and Fire Truck (Regimen [R1]); the unfilled bars indicate subjects who received training for the signs of Crosswalk, Railroad Crossing, and Ambulance (Regimen 2 [R2]). Error bars indicate *SE*s.

We conducted an ANOVA that excluded untrained signs and focused instead on the effects of Memoranda (three vs. six), along with the effects of Multi-Tasking. As illustrated in Figure 6, there was no significant effect of Multi-Tasking, *F*(2, 78) = 2.81, *p* = 0.07 (η_*p*_^2^ = 0.07), no effect of Memoranda, *F*(2, 39) = 0.25, *p* = 0.78 (η_*p*_^2^ = 0.01), and no interaction between the two factors, *F*(4, 78) = 0.87, *p* = 0.49 (η_*p*_^2^ = 0.04).

### Perceived Immediacy

**Figure 7:**
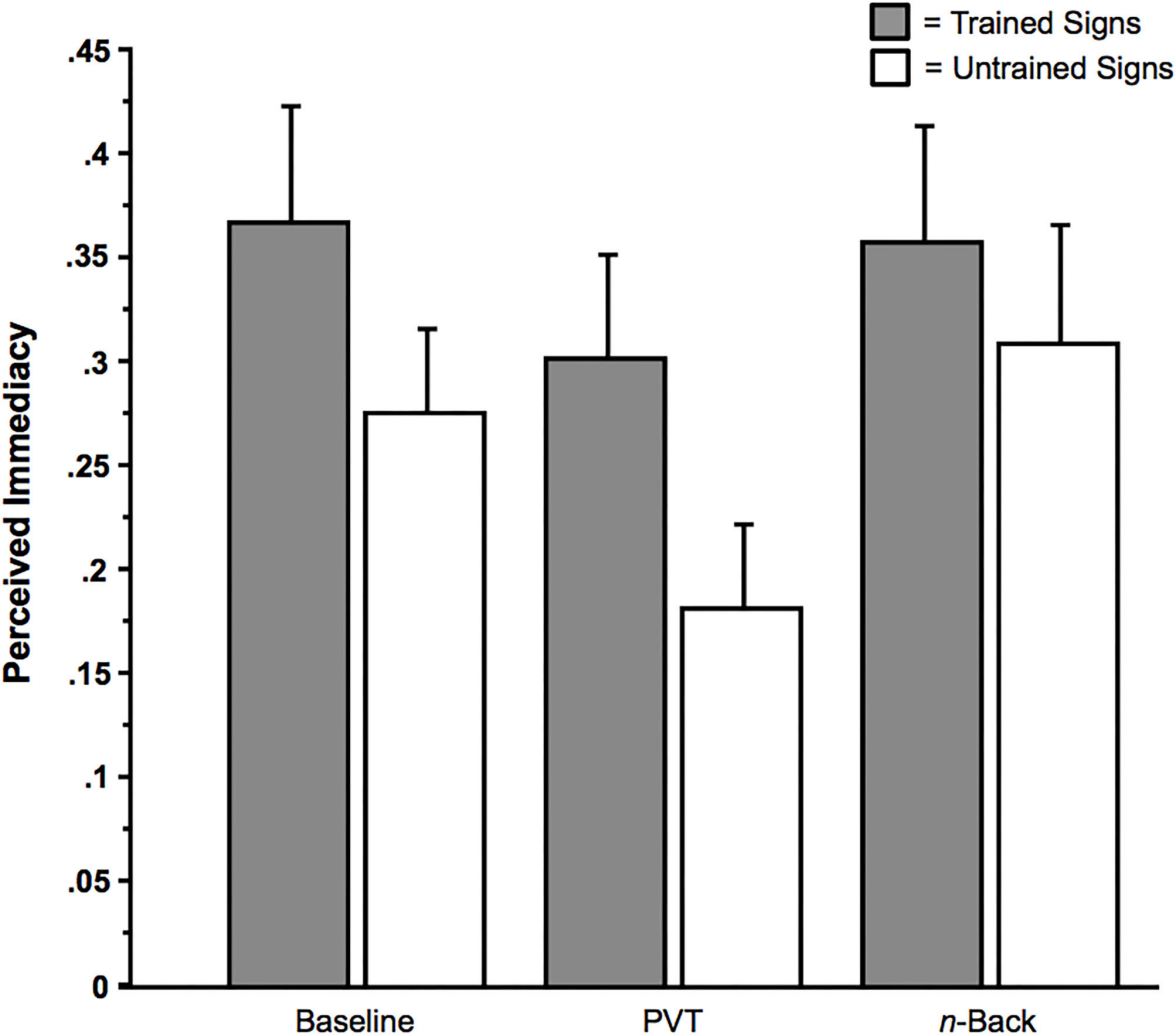
Perceived immediacy of involuntary imagery as a function of Sign Type (Trained vs. Untrained [Control]) and Multi-Tasking (None [Baseline], Psychomotor Vigilance Task [PVT], or *n*-back). Error bars indicate *SE*s.

When subjects rated the immediacy with which the sign-related imagery was experienced, in response to the question, “*If you did experience verbal imagery, did the words come to mind immediately? (Yes or No?),”* there was only an effect of Sign Type (Trained or Untrained). In a 2 × 3 ANOVA with the within-subjects factor Sign Type (Trained or Untrained), and the within-subjects factor Multi-Tasking (None [Baseline], PVT, or *n*-back), there was only a main effect of Sign Type, *F*(1, 27) = 6.51, *p* = 0.017 (η_*p*_^2^ = 0.19), in which the involuntary imagery from trained signs was more likely to be perceived as immediate than that from untrained signs (Figure 7). There were no effects of Multi-tasking, Memoranda (six signals vs. three signals), or any interactions among these factors, *F*s < 3.09, *p*s > 0.05.

### Action-Related Urges

We examined urges to press buttons during the Suppress condition, in response to the question, “*During these last moments (5s) of the video clip, how strong was the urge to press a button (8-point scale)?”* The only noteworthy effect is the following. In an ANOVA that excluded untrained signs and focused instead on the effects of Memoranda (three vs. six) and the effects of Multi-Tasking, there was an unpredicted effect of Multi-Tasking on these urges, *F*(2, 78) = 3.77, *p* = 0.03 (η_*p*_^2^ = 0.09). This effect, and what was observed regarding performance in the Respond condition (discussed next), will require further investigation. See Table 1 for descriptive statistics for the conditions of the Suppress condition.

**Table 1:** Descriptive statistics for conditions of the suppress condition, including the psychomotor vigilance task (PVT): Means per condition with *SD*s presented in parentheses.

	Involuntary sign imagery^*^	**Immediacy**	**Urges to err**
**Three signs trained**			
Baseline	0.31 (0.26)	0.37 (0.29)	2.24 (1.67)
*n-*back	0.28 (0.28)	0.36 (0.30)	2.61 (1.81)
PVT	0.25 (0.23)	0.30 (0.27)	2.80 (1.91)
**Six signs trained**			
Baseline	0.36 (0.24)	0.41 (0.25)	2.56 (1.70)
*n-*back	0.29 (0.19)	0.36 (0.24)	2.91 (1.97)
PVT	0.31 (0.27)	0.38 (0.26)	2.94 (1.85)
**Untrained signs**			
Baseline	0.21 (0.17)	0.28 (0.21)	2.25 (1.69)
*n-*back	0.13 (0.20)	0.31 (0.30)	2.57 (1.89)
PVT	0.16 (0.21)	0.18 (0.21)	2.58 (1.77)

* *For the involuntary sign imagery, each of the mean proportions is significantly different from zero, ps < 0.05. The same pattern of results is found with arcsine transformations of the proportions*.

### Behavioral Performance and Engagement in Secondary Tasks

**Figure 8:**
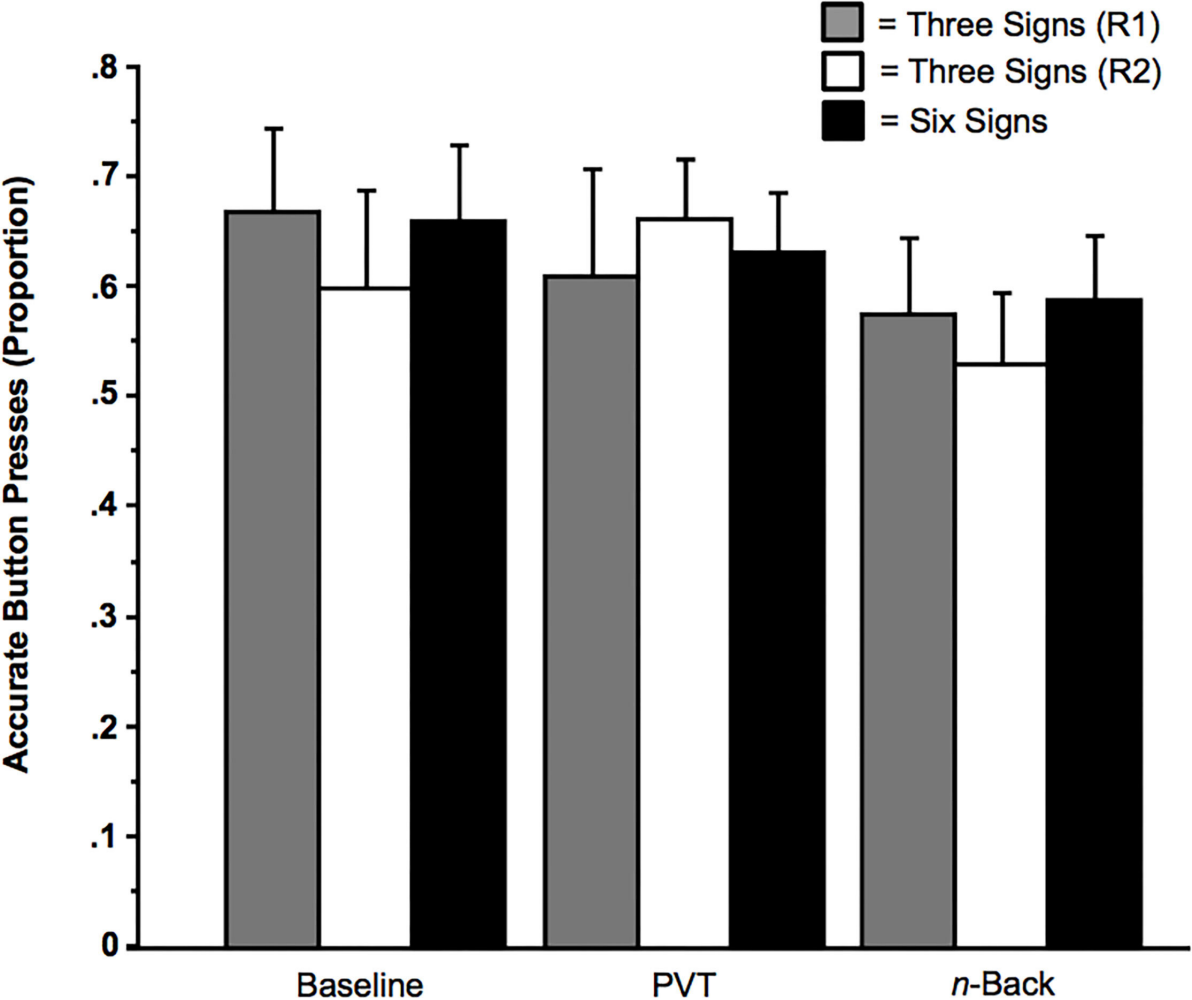
Correct button press to presence of trained sign as a function of Memoranda (three signals or six signals) and Multi-Tasking (None [Baseline], Psychomotor Vigilance Task [PVT], or *n*-back). The solid black bars indicate subjects who received training for all six signs. The gray bars indicate subjects who received training for the three signs of Bike Lane, Speed Bump, and Fire Truck (Regimen [R1]); the unfilled bars indicate subjects who received training for the signs of Crosswalk, Railroad Crossing, and Ambulance (Regimen 2 [R2]). Error bars indicate *SE*s.

Computer malfunctions and script errors led to the loss of 9 (0.004%) of 2,460 trials (from 41 subjects) in the Respond condition. The mean removal of trials per subject was <1 trial (*M* = 0.22). These malfunctions caused some critical trials to appear more than once per session. Data from these repeated stimuli were removed from all analyses. Consistent with the data from the Suppress condition, subjects were sensitive to the trained signs. As revealed in Figure 8, accuracy was significantly above chance levels (with chance levels being 0.33 for the memory load of three signs and being 0.17 for the memory load of six signs), *t*s > 2.88, *p*s < 0.014. Moreover, consistent with the data from the Suppress condition, and as revealed in Figure 8, the factors of Memoranda (three signals vs. six signals) and of Multi-Tasking had no effect on the rate of responding to the trained signs, *F*s < 2.40, *p*s > 0.10. Moreover, these factors had no main effects or interaction effects on RTs, *F*s < 3.24, *p*s ≥ 0.05.

As illustrated in Figure 5, involuntary sign-related imagery arose even when subjects were multi-tasking (i.e., concurrently performing the PVT or *n*-back). There is evidence that the subjects were indeed engaged in these secondary tasks. Regarding the *n*-back, subjects performed accurately (i.e., pressing the button when there was a repeated number and *not* pressing the button when there was no repeated number) on a proportion of 0.87 of the trials (*SD* = 0.13, Range = 0.45 to 1). Regarding the PVT, subjects correctly pressed the button in response to the sound of the beep on a proportion of 0.99 of the critical trials (*SD* = 0.02, Range = 0.90 to 1.00), with a mean response latency of 802.15 ms (*SD* = 262.26 ms, Range = 333.80 to 1,447.85 ms).

## General Discussion

In interference paradigms, distractors can activate urges, action dispositions, and mental imagery. Can such stimuli, when embedded in a dynamic and ecologically-valid stimulus scene, influence conscious processes in a similar manner? Specifically, can signs embedded in video footage of real street scenes trigger involuntary action-related imagery? The data from our project suggest that the answers to these questions is yes.

Our data reveal that involuntary imagery can arise in a substantive manner by stimuli (signs) that are embedded in dynamic video footage that has high ecological validity. It is noteworthy that such imagery arose despite the intentions of the subject, the complexity of the stimulus scene, and the minimal amount of training, which consisted of only 10 trials. The effect size of the involuntary imagery was comparable to that of other studies designed to illuminate the boundary conditions of such involuntary phenomena (e.g., Bui et al., 2019; Cushing et al., 2019). In addition, the data revealed that the task we developed is engaging at an informative level, with the task not being too easy (no ceiling effects) or too challenging (no floor effects).

One aim of the data analysis was to ascertain whether involuntary imagery arises in a predictable manner from the video clips we developed. The stimuli were developed from over 36 h of actual driving footage. These stimuli (i.e., the signs) that were embedded in the video footage were designed to appear within a dynamic and ecologically-valid context. Despite this and the intentions of the subjects, involuntary imagery arose in response to the signs on a substantive proportion of the trials (*M*_Baseline Condition =_ 0.31 of 20 trials). This effect is noteworthy because subjects were instructed: “*IMPORTANT: During the task, please DO NOT respond to any of the signs… Also, try to NOT think of the response you learned to any of the signs*.”

The data support the hypothesis that these forms of involuntary imagery are robust and will arise even under different conditions of Memoranda (e.g., from the training of six critical signs instead of just three critical signs) and under conditions of multi-tasking (e.g., secondary tasks such as the PVT or *n*-back). Consistent with theorizing concerning the prepared reflex (Exner, 1879; Ach, 1905/1951; Woodworth, 1939; Gollwitzer, 1999; Hommel, 2000; Cohen-Kdoshay and Meiran, 2009; Cole et al., 2013; Pereg and Meiran, 2019), the manipulations of memoranda size and of multi-tasking did not seem to diminish substantively the rates of involuntary imagery. Theories concerning the nature of cognitive resources must take into account such an observation and also the more general notion of the prepared reflex, a mental act that seems to somehow be unaffected by the taxing of cognitive resources (Ach, 1905/1951; Gollwitzer, 1999; see Pereg and Meiran, 2019). It has been proposed that these effects, stemming from prepared reflexes, are often insuppressible and *motivation-independent* (Gollwitzer et al., 2009), requiring only the pre-stimulus activation of the appropriate action set (e.g., by external stimuli, task instructions, or prospective memory). The mental imagery experienced by our subjects, as fleeting as it might have been, is a case of high-level cognition, a phenomenon that requires at least some cognitive resources. Theories concerning cognitive resources need to account for observations in which such cognitions, and the kinds of cognitive effects trigged by prepared reflexes, are somehow unperturbed by, for example, dual-task conditions.

It is important to add that the data are based on subjects' self-reports of the conscious contents that were experienced after the presentation of the sign. Such self-reports, occurring moments after the experience of the relevant conscious experience, can be inaccurate as a result of (a) inaccurate memories of fleeting conscious contents (Block, 2007), or (b) subjects basing their reports on a strategy of how to comport oneself during an experiment (see discussion in Morsella et al., 2009). Given the conscientiousness of the subjects, as displayed, for example, in their accuracy rates on the two secondary tasks, we do not believe that subjects were confabulating or inaccurate about their introspections.

The present research has implications for the emerging technologies associated with semi-automated driving. The safe “intelligent interaction” between driver and vehicle requires that the communicative signals from vehicle to driver be as effective as possible at activating the appropriate cognitions, mental imagery, and behavioral inclinations (e.g., urges), even when (*a*) the driver is engaged in a secondary task and (*b*) such inclinations should *not* be expressed behaviorally in a particular context (e.g., because of the task set; Morsella et al., 2012). While monitoring the navigation of a semi-automated vehicle, a driver must remain sensitive to important signals and stimuli coming from outside of the vehicle (e.g., sirens and a school zone sign). The “stimulus control” exhibited by the trained signs in our project, which stemmed in part from the training session, provides a possible way in which these important signals could be more effective at influencing a driver's awareness and action selection. Thus, perhaps these initial data provide some evidence that a technique similar to that of our training session can benefit drivers' responses, especially in a scenario such as that of semi-automated driving, in which the driver is presented with more than a handful of signals and stimuli from both within the vehicle and from outside of the vehicle (Green et al., 1993).

To conclude, the distractor-elicited involuntary imagery that is observed in laboratory response interference paradigms does appear to arise in highly ecologically-valid conditions involving complex and dynamic stimuli (e.g., simulations of semi-automated driving experiences).

## Data Availability Statement

The original contributions presented in the study are included in the article/supplementary material, further inquiries can be directed to the corresponding author/s.

## Ethics Statement

All studies involving human participants were reviewed and approved by Internal Review Committee, San Francisco State University, 1600 Holloway Ave, Administration 471, San Francisco, CA 94132. The participants provided their written informed consent to participate in this study.

## Author Contributions

AV conducted the extensive piloting that was necessary for developing the experimental paradigm. AV was in charge of all the data collection. EM developed all the video stimuli. AV and EM conducted the data analyses and crafted the first, rough versions of the manuscript. HT provided critical feedback during each stage of the research process, from study designing to the writing of the manuscript. All authors contributed to the design of the study and the writing of the manuscript.

## Funding

This study received funding from the Toyota Collaborative Safety Research Center. The funder was not directly involved in the study design, the collection, analysis, interpretation of data, the writing of this article or the decision to submit it for publication.

## Conflict of Interest

HT was employed by the company Toyota Motor North America, Inc. The remaining authors declare that the research was conducted in the absence of any commercial or financial relationships that could be construed as a potential conflict of interest.

## Publisher's Note

All claims expressed in this article are solely those of the authors and do not necessarily represent those of their affiliated organizations, or those of the publisher, the editors and the reviewers. Any product that may be evaluated in this article, or claim that may be made by its manufacturer, is not guaranteed or endorsed by the publisher.

## Footnotes

^1^In the Stroop task (Stroop, 1935), participants are instructed to name the color in which a word or string of letters is presented. Interference arises in the Incongruent condition, in which, for example, the word “BLUE” is presented in an incongruent color such as red (see review of the Stroop task in MacLeod and MacDonald, 2000).

^1^Several theorists have attributed RI to the automatic, training-based activation of response codes by distractors (Eriksen and Schultz, 1979; Coles et al., 1985). The developers of the flanker task have explained this activation, which is mediated unconsciously, by appealing to the notion of “continuous flow” (Eriksen and Schultz, 1979), a notion that is based on observations of the basic neurophysiological characteristics of perceptual processing (Ganz, 1975). Continuous flow is similar to the notion of “cascade processing” in *parallel distributed processing* (PDP) models (McClelland, 1979). From the standpoint of continuous flow, once there is an association formed between percepts and response codes, then activation cannot help but flow from one stage of processing (e.g., the perceptual stage) to the next stage (e.g., motor preparation). Accordingly, psychophysiological research shows that, in a response interference task such as the flanker task, competition involves the simultaneous activation of the neural processes associated with the target-related responses and the distractor-related responses (DeSoto et al., 2001).

^2^These self-reported urges to make a mistake (“urges to err,” for short) are measured after each trial by asking subjects, “How strong was your urge to make a mistake?” Subjects respond by using an 8-point scale, in which 1 signifies “almost no urge” and 8 signifies “extremely strong urge.”

^3^Subjects read the following instructions, which were based on Jantz et al. (2014). “*In this task, you will be asked to report about the occurrence of verbal imagery. ‘Verbal imagery’ refers to the following. Without saying it aloud, take a moment to imagine what the word ‘HOUSE’ sounds like. Take a moment to imagine what the word ‘FLOWER’ sounds like. You have just experienced an example of auditory verbal imagery. Another example of verbal imagery would be if you heard ‘Mary had a little…’ and then heard in your mind ‘lamb’. The word ‘lamb’ would be a case of verbal imagery.”* On a second screen, it was further clarified that, “*Sometimes you experience verbal imagery, as when you are holding a number you just heard in mind long enough to dial it. Sometimes you don't experience verbal imagery. Verbal imagery is basically words heard only in your mind but not aloud, as when you quietly rehearse a poem or rehearse the words to a song. For this task, report only imagery that you are comfortable sharing with a stranger. If you do not understand what is meant by ‘verbal imagery,’ please contact the experimenter for further clarification.”*

^4^The results of this analysis were complemented by the data that emerged from the first question after each critical trial, “*Did you just experience any verbal imagery? That is, did you experience any verbal imagery during the last moments (that is, the last five seconds) of the video? (Yes or No?)*.” Again, there was only a main effect of Sign Type (Trained vs. Untrained), *F*(1, 26) = 8.20, *p* = *0*.008 (η_*p*_^2^ = 0.24), and no other main effects or interaction effects, *F*s < 2.00, *p*s > 0.14.

## Appendix

For the crosswalk sign, the instructions were “*When you see a CROSSWALK sign, say ‘Yellow Yield’ aloud and press the YELLOW button. It is important that you respond as fast and as accurately as possible. Your hands must be in ‘rest position.’ Press G when you are ready to see the sign and respond to it.”*

For the bike lane sign, the instructions were “*When you see a BIKE LANE sign, say ‘Green Scan’ aloud and press the GREEN button. It is important that you respond as fast and as accurately as possible. Your hands must be in ‘rest position.’ Press G when you are ready to see the sign and respond to it.”*

For the speed bump sign, the instructions were “*When you see a SPEED BUMP sign, say ‘Orange Slow’ aloud and press the ORANGE button. It is important that you respond as fast and as accurately as possible. Your hands must be in ‘rest position.’ Press G when you are ready to see the sign and respond to it.”*

For the railroad crossing sign, the instructions were “*When you see a RAILROAD CROSSING sign, say ‘Purple Pause’ aloud and press the PURPLE button. It is important that you respond as fast and as accurately as possible. Your hands must be in ‘rest position.’ Press G when you are ready to see the sign and respond to it.”*

For the fire truck sign, the instructions were “*Red lines on the top of the screen indicate the presence of a fire truck. When you see the FIRE TRUCK sign, say ‘Red Move’ aloud and press the RED button. It is important that you respond as fast and as accurately as possible. Your hands must be in ‘rest position.’ Press G when you are ready to see the sign and respond to it.”*

For the ambulance sign, the instructions were “*Blue lines on the top of the screen indicate the presence of an ambulance. When you see the AMBULANCE sign, say ‘Blue Hear’ aloud and press the BLUE button. It is important that you respond as fast and as accurately as possible. Your hands must be in ‘rest position.’ Press G when you are ready to see the sign and respond to it.”*
